# Risk Models for Advanced Melanoma Patients Under Anti-PD-1 Monotherapy—*Ad hoc* Analyses of Pooled Data From Two Clinical Trials

**DOI:** 10.3389/fonc.2021.639085

**Published:** 2021-05-20

**Authors:** Xue Bai, Jie Dai, Caili Li, Chuanliang Cui, Lili Mao, Xiaoting Wei, Xinan Sheng, Zhihong Chi, Xieqiao Yan, Bixia Tang, Bin Lian, Xuan Wang, Li Zhou, Siming Li, Yan Kong, Zhonghui Qi, Huayan Xu, Rong Duan, Jun Guo, Lu Si

**Affiliations:** ^1^Key Laboratory of Carcinogenesis and Translational Research (Ministry of Education, Beijing), Department of Melanoma, Peking University Cancer Hospital and Institute, Beijing, China; ^2^Key Laboratory of Carcinogenesis and Translational Research (Ministry of Education, Beijing), Department of Genitourinary Cancers, Peking University Cancer Hospital and Institute, Beijing, China; ^3^Key Laboratory of Carcinogenesis and Translational Research (Ministry of Education, Beijing), Department of Melanoma, Peking University Cancer Hospital & Institute, Beijing, China; ^4^Key Laboratory of Carcinogenesis and Translational Research (Ministry of Education, Beijing), Department of Genitourinary Cancers, Peking University Cancer Hospital & Institute, Beijing, China

**Keywords:** PD-1, melanoma, progression free survival, overall survival, best response, risk model

## Abstract

**Background:** The best response and survival outcomes between advanced melanoma patients treated with the anti-PD-1 monotherapy vary greatly, rendering a risk model in need to optimally stratify patients based on their likelihood to benefit from the said treatment.

**Methods:** We performed an *ad hoc* analysis of 89 advanced melanoma patients treated with the anti-PD-1 monotherapy from two prospective clinical trials at the Peking University Cancer Hospital from April 2016 to May 2018. Clinicodemographical characteristics, baseline and early-on-treatment (median 0.6 months after anti-PD-1 monotherapy initiation) routine laboratory variables, including complete blood count and general chemistry, and best response/survival data were extracted and analyzed in both univariate and multivariate logistic and Cox proportional hazard models.

**Results:** After three rounds of screening, risk factors associated with a poorer PFS included a high pre-treatment neutrophil, derived neutrophil-lymphocyte ratio (dNLR), low pre-treatment hemoglobin, and low early-on-/pre-treatment fold change of eosinophil; those with a poorer OS included a high pre-treatment neutrophil, eosinophil, PLT, early-on/pre-treatment fold change of LDH and neutrophil; and those with a poorer best response included a high pre-treatment NLR and early-on-/pre-treatment LDH fold change. Risk models (scale: low, median-low, median high, and high risk) were established based on these risk factors as dichotomous variables and M stage (with vs. without distant metastasis) for PFS (HR 1.976, 95% CI, 1.507–2.592, *P* < 0.001), OS (HR 2.348, 95% CI, 1.688–3.266), and non-responder (OR 3.586, 95% CI, 1.668–7.713, *P* = 0.001), respectively. For patients with low, median-low, median-high, and high risks of developing disease progression (PD), six-month PFS rates were 64.3% (95% CI, 43.5–95.0%), 37.5% (95% CI, 22.4–62.9%), 9.1% (95% CI, 3.1–26.7%), and 0%, respectively. For patients with OS risks of low, median-low, median-high, and high, OS rates at 12 months were 82.5% (95% CI, 63.1–100%), 76.6% (95% CI, 58.4–100%), 42.1% (95% CI, 26.3–67.3%), and 23.9% (95% CI, 11.1–51.3%), respectively. For patients with risks of low, median-low, median-high, and high of being a non-responder, objective response rates were 50.0% (95% CI, 15.7–84.3%), 27.8% (95% CI, 9.7–53.5%), 10.3% (95% CI, 2.9–24.2%), and 0%, respectively.

**Conclusion:** A risk scoring model based on the clinicodemographical characteristics and easily obtainable routinely tested laboratory biomarkers may facilitate the best response and survival outcome prediction and personalized therapeutic decision making for the anti-PD-1 monotherapy treated advanced melanoma patients in Asia.

## Introduction

Anti-programmed cell death protein-1 (PD-1) antibodies, as monotherapy, has been recently proved efficacious (1–3) and approved by China FDA in advanced melanoma patients. Although a substantial survival benefit has been brought in, a vast majority of patients develop resistance to the anti-PD-1 monotherapy (4–7). Ever since, there have been tremendous efforts in developing biomarkers to facilitate clinical decision making. Although tumor mutational burden (TMB) (8), the expression of programmed cell death protein ligand-1 (PD-L1) (9), tumor infiltrating lymphocytes (TILs) (10), and several melanoma/immune-pertinent signatures (11–14) have been reported to be positively correlated with a therapeutic benefit from anti-PD-1 monotherapy, these are tumor sample-based, thus, requiring invasive biopsy/surgical procedures and are also computationally demanding, which render them less feasible to be adopted in the daily clinical practice. Accumulating data has demonstrated that simple complete blood count and general chemistry results may be informative with regard to therapeutic outcomes of advanced melanoma under the anti-PD-1 monotherapy (15–20), making them as promising collection of candidates for a potential biomarker panel development.

Noticeably, emerging data has shown that there is a high degree of heterogeneity with regard to the therapeutic response to the anti-PD-1 monotherapy between patients with different subtypes of advanced melanomas. Namely, acral and mucosal melanomas which dominate in Asia (21) had poorer clinical outcomes compared to the predominant cutaneous subtype in both the US and Europe due to a lower tumor mutational burden and higher proportion of chromosome structural variations (22). Yet, there is no biomarker panel available for this subgroup of patients who are underrepresented in previous phase III randomized control trials that led to the FDA approval of pembrolizumab and nivolumab (4, 5).

To solve this clinically relevant issue, we performed *ad hoc* analyses to developed risk models to predict the progression-free survival (PFS), overall survival (OS), and response, respectively, based on the clinicodemographical characteristics and already available pre- and early-on treatment routinely tested laboratory parameters from the pooled data of two clinical trials testing anti-PD-1 monotherapies in advanced melanoma patients in China to facilitate future patient screening and clinical therapeutic decision making.

## Methods

### Patients

Single center patient data from Peking University Cancer Hospital was pooled from two clinical trials (NCT02821000 and NCT02738489) testing anti-PD-1 monotherapies in advanced melanoma. The following clinical data was collected: baseline demographics, melanoma pertinent information [subtype, mutational status, stage, previous treatment(s)], survival outcomes (both PFS and OS), best response per Response Evaluation Criteria in Solid Tumors version 1.1 (RECIST v1.1), and routine laboratory test results (complete blood count and general chemistry) at the time points of both pre- and early-on-anti-PD-1 monotherapy (first blood draw per trial protocol, median 0.6 months after anti-PD-1 monotherapy initiation). The following data was collected from the general chemistry test result: lactate dehydrogenase (LDH), total protein, albumin, globulin, albumin/globulin ratio (A/G) and glucose. The data drawn from the complete blood count results included white blood cell (WBC), red blood cell (RBC), hemoglobin (Hb), platelet (PLT), neutrophil, lymphocyte, neutrophil/lymphocyte ratio (NLR), derived NLR [neutrophil/(WBC-lymphocyte)], monocyte, eosinophil, and basophil.

### Statistical Methods

Continuous variables of all clinicodemographical and pre- and on-anti-PD-1 monotherapy laboratory test results were first screened via the Cox proportional hazard regression model, using a *P* < 0.25 as the threshold for PFS, OS, and best response (responder vs. non-responder). For each candidate that passed the initial screening process, an optimal cutoff value was calculated by the R-based Maxstat and optimalCutpoints packages for survival outcomes and best response, respectively. For each parameter, different cut-off values were applied. Each corresponding log-rank (for survival analysis using Cox proportional hazard regression model) or specificity/sensitivity (for best response analysis using logistic regression model) statistics were calculated, and then the cut-off value that maximized the log-rank or Youden Index was chosen as the optimal threshold for survival and best response outcomes, respectively. Continuous variables were dichotomized into high (> threshold) and low (≤ threshold) categories using the above-determined optimal cutoff values, and then tested using the univariate Cox proportional hazard regression model or logistic regression model for survival outcomes and best response, respectively. Also tested were other binary clinicodemographical variables, including sex, melanoma subtypes (cutaneous vs. non-cutaneous), with vs. without BRAF V600 mutation/prior systemic anti-tumor therapy. Those with a *P* < 0.05 were incorporated into the multivariate Cox proportional hazard regression and logistic models for survival outcomes (both PFS and OS) and response, respectively. These binary parameters with a *P* < 0.10 in the multivariate analysis were included in the risk model, scoring 0 or 1, above or equal/below each threshold, depending on its association with survival outcomes, and also included was the M stage (with vs. without distant metastasis). The sum of the score of each parameter was used to determine the risk stratification that each patient belonged to.

All statistical tests were two sided with a *P* < 0.05 as statistically significant. All statistical analyses were performed using R (version 3.6.0, R packages maxstat, ggplot2, ggpubr, survival, optimalcutpoints, and survminer).

## Results

In total, 89 patients were included in this *ad hoc* pooled analysis. The median and minimum follow-up were 22.8 and 6.4 months, respectively. Median PFS was 2.9 months (95% CI, 2.7–4.4) and median OS was 17.0 months (95% CI, 12.7–27.6). Per RECIST v1.1, there were 13 (14.6%) responders (with complete remission or partial remission as their best response) and 73 (85.4%) non-responders. Median age of the patients was 53 years old (range 27–78), and 44 (49.4%) were male. In terms of the melanoma subtype distribution, there were 19 (21.3%) patients with cutaneous melanoma, 37 (41.6%) with acral, and 18 (20.2%) with mucosal melanoma. Thirty-three (37.1%) patients were of stage M1a disease, 23 (25.8%) of M1b, 27 (30.3%) of M1c, and 5 (5.6%) of M1d. Detailed clinicodemographical characteristics of the patients are listed in Supplementary Table 1.

### Biomarker Screening for PFS

The continuous variables were first screened using the univariate Cox proportional hazard regression model, with a *P* < 0.25 as the threshold. The parameters that passed the first round of screening process included age (*P* = 0.09); pre-treatment parameters including albumin (*P* = 0.04), globulin (*P* = 0.09), A/G (*P* = 0.10), neutrophil (*P* = 0.02), NLR (*P* = 0.09), dNLR (*P* = 0.06), WBC (*P* = 0.07), Hb (*P* = 0.06), and PLT (*P* = 0.007); and early-on-/pre-treatment fold change parameters including LDH (*P* = 0.24), eosinophil (*P* = 0.05), and RBC (*P* = 0.22). Then, the optimal cut off value for the dichotomization of each of these continuous variables were calculated to maximize statistical significance by the Log-rank test. Details are listed in Supplementary Table 2.

These newly dichotomized variables (high vs. low) were then subjected to the second round of screening using the univariate Cox proportional hazard regression model, together with other clinicodemographical binary variables. Details are shown in Supplementary Table 3. Those with a *P* < 0.05 were thus incorporated into the multivariate analysis, which included age (threshold 55 years old); pre-treatment parameters including albumin (threshold 42.5 g/L), neutrophil (threshold 4.11^*^10^9^/L), NLR (threshold 1.99), dNLR (threshold 0.9), Hb (153 g/L), and PLT (threshold 226^*^10^9^/L); and early-on-/pre-treatment parameters including eosinophil (threshold 1.176) and RBC (threshold 0.940). Details are listed in Supplementary Tables 3, 4.

### Risk Model for PFS

**Figure 1:**
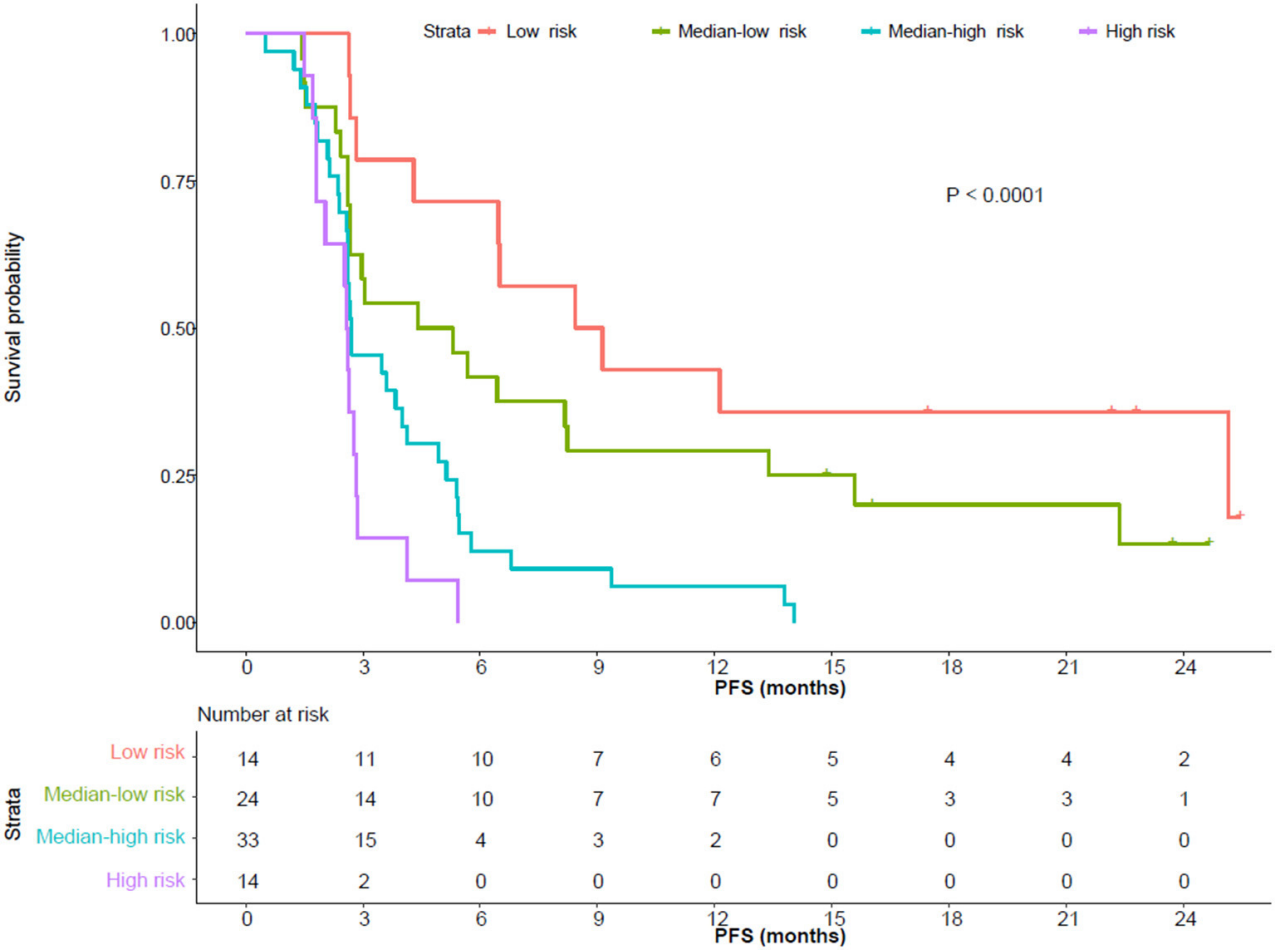
Progression-free survival (PFS) of patients with different risks. The median PFS of patients with low, median-low, median-high, and high risks was 8.8 (95% CI, 6.5-not reached), 4.9 (95% CI, 2.7–13.4), 2.7 (95% CI, 2.6–4.9) and 2.6 (95% CI, 2.0–4.1) months, respectively.

The parameters with *P* < 0.10 in the multivariate Cox proportional hazard regression model (Supplementary Table 4) and known prognostic factor M stage were incorporated in the risk model for PFS included. In total, five risk factors were included. Each status that was negatively correlated with PFS scored 1, namely pre-treatment neutrophil (>4.11^*^10^9^/L), dNLR (>0.9), Hb (≤ 153 g/L), early-on-/pre-treatment fold change of eosinophil (≤ 1.176), and distant metastasis; otherwise scored 0. Patients who scored 0–1, 2, 3, and 4–5 were categorized as low, median-low, median-high, and high risk subgroups (scores 0/1 and 4/5 combined due to the limited sample size). The higher the risk group the patient belonged to, the more likely he/she was to develop disease progression (HR 1.976, 95% CI, 1.507–2.592, *P* < 0.001). The 6-month PFS rate of the patients with a low, median-low, median-high, and high risk of developing disease progression was 64.3% (95% CI, 43.5–95.0%), 37.5% (95% CI, 22.4–62.9%), 9.1% (95% CI, 3.1–26.7%), and 0%, respectively. Details are in Table 1 and Figure 1.

**Table 1:** Risk model for PFS (univariate Cox proportional hazard regression model, *P* < 0.001).

			**PFS rate(%)**		
**Risk**	**Score**	**Median PFS** **(month, 95% CI)**	**3-month** **(95% CI)**	**6-month** **(95% CI)**	**12-month** **(95% CI)**
Low (*n* = 14)	0–1	8.8 (6.5–NR)	71.4 (51.3–100)	64.3 (43.5–95.0)	35.7 (17.7–72.1)
Median-low (*n* = 24)	2	4.9 (2.7–13.4)	54.2 (37.5–78.3)	37.5 (22.4–62.9)	25.0 (12.5–50.0)
Median-high (*n* = 33)	3	2.7 (2.6–4.9)	42.4 (28.5–63.1)	9.1 (3.1–26.7)	3.0 (0.4–21.0)
High (*n* = 14)	4–5	2.6 (2.0–4.1)	7.1 (1.1–47.2)	0	0

*NR, not reached. Scores 0/1 and 4/5 are converged due to the limited sample size*.

### Biomarker Screening for OS

The same method was applied to screen the candidate biomarkers of OS. The continuous variables that survived the first round of screening included pre-treatment LDH (*P* = 0.20), neutrophil (*P* = 0.003), NLR (*P* = 0.01), monocyte (*P* = 0.15), eosinophil (*P* = 0.11), WBC (*P* = 0.01), and PLT (*P* = 0.04); early-on-/pre-treatment fold change of LDH (*P* = 0.04), neutrophil (*P* = 0.14), dNLR (*P* = 0.17), monocyte (*P* = 0.18), and WBC (*P* = 0.20). A threshold for the dichotomization that maximizes the statistical significance of OS using the Log-rank test was then calculated for each candidate. Details are listed in Supplementary Table 5.

Dichotomized parameters and other binary clinicodemographical variables were then assessed via the univariate Cox proportional hazard regression model (details are listed in Supplementary Table 6). Those that were significantly associated with OS were selected to be incorporated in the multivariate analysis, which included pre-treatment neutrophil (>5.16^*^10^9^/L), NLR (>4.33), monocyte (>0.47^*^10^9^/L), eosinophil (>0.09^*^10^9^/L), WBC (>4.29^*^10^9^/L), and PLT (>226^*^10^9^/L), and early-on-/pre-treatment fold change of LDH (>0.911) and neutrophil (>1.539) (Supplementary Table 7).

### Risk Model for OS

**Figure 2:**
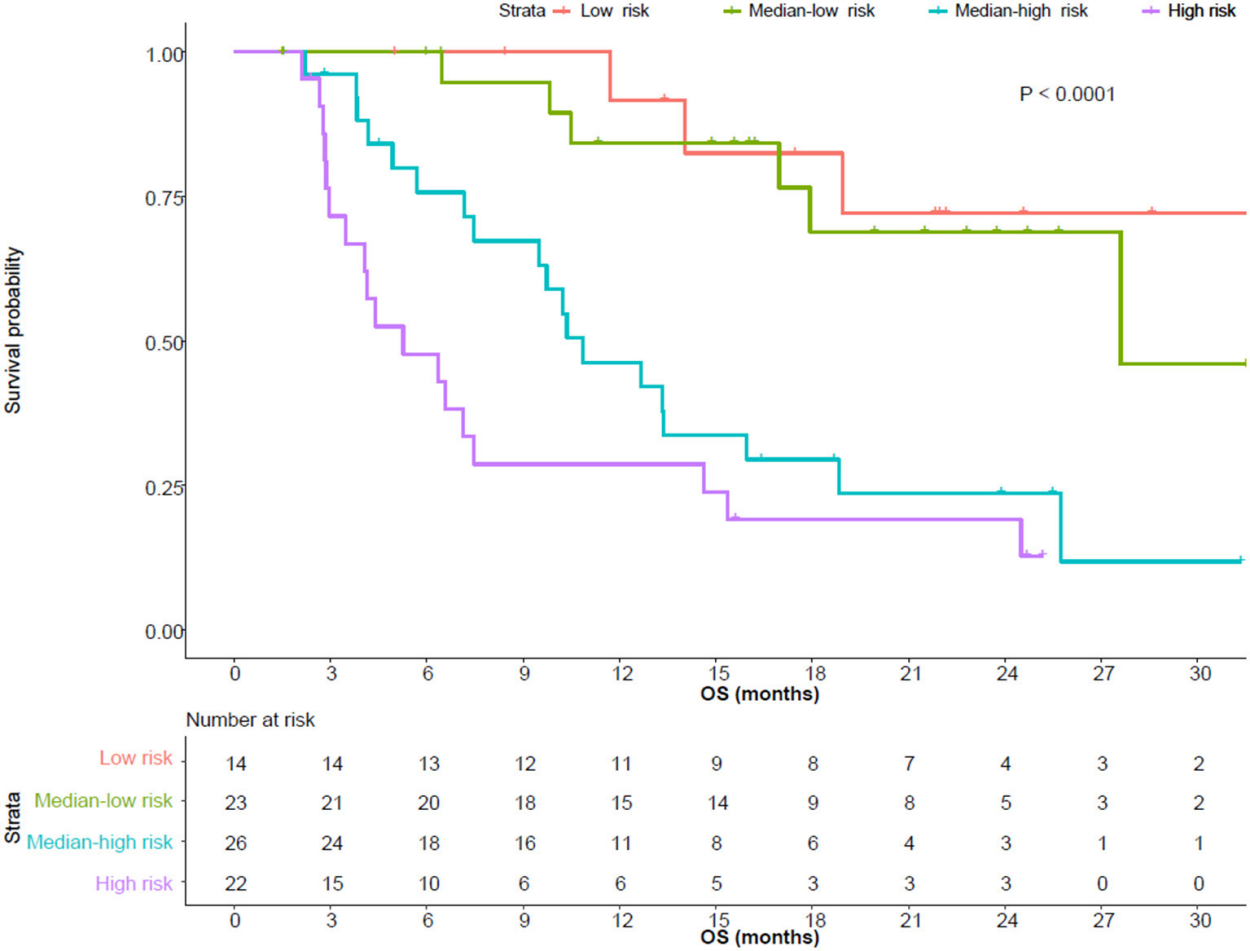
Overall survival (OS) of patients with different risks. The median OS of patients with low, median-low, median-high, and high risks was not reached (95% CI, 18.9-not reached), 27.6 (95% CI, 17.9-not reached), 10.9 (95% CI, 9.5-not reached) and 5.3 (95% CI, 3.5–15.4) months, respectively.

Same screening criteria were adopted for the building of the OS risk model as for PFS. In total, six parameters were included in the risk model. Same as in the PFS risk model, each status which was negatively correlated with OS scored 1, including pre-treatment neutrophil (>5.16^*^10^9^/L), eosinophil (>0.09^*^10^9^/L), PLT (>226^*^10^9^/L), early-on/pre-treatment fold change of LDH (>0.911), neutrophil (>1.539), and distant metastasis; otherwise, 0. Patients who scored 0–1, 2, 3, and 4–5 were sub-grouped into low, median-low, median-high, and high risk categories (scores 0/1 and 4/5 combined due to the limited sample size). The higher the risk category the patient belonged to, the more likely he/she developed an OS event (HR 2.348, 95% CI, 1.688–3.266). The 12-month OS rate of the patients with a low, median-low, median-high, and high risk of developing OS event was 82.5% (95% CI, 63.1–100), 76.6% (95% CI, 58.4–100%), 42.1% (95% CI, 26.3–67.3%), and 23.9% (95% CI, 11.1–51.3%), respectively. Details are in Table 2 and Figure 2.

**Table 2:** Risk model for OS (univariate Cox proportional hazard regression model, *P* < 0.001).

			**OS rate(%)**		
**Risk**	**Score**	**Median OS (month, 95% CI)**	**6-month (95% CI)**	**12-month (95% CI)**	**24-month (95% CI)**
Low (*n* = 14)	0–1	NR (18.9–NR)	100	82.5 (63.1–100)	72.2 (49.6–100)
Median-low (*n* = 23)	2	27.6 (17.9–NR)	94.7 (85.2–100)	76.6 (58.4–100)	45.9 (19.3–100)
Median-high (*n* = 26)	3	10.9 (9.5–NR)	71.5 (55.7–91.9)	42.1 (26.3–67.3)	11.8 (2.4–57.2)
High (*n* = 22)	4–5	5.3 (3.5–NR)	43.0 (26.2–70.3)	23.9 (11.1–51.3)	12.7 (3.9–41.8)

*NR, not reached*.

### Biomarker Screening for Response (Responder vs. Non-responder)

The continuous variables were first screened using the univariate logistic regression model, with a *P* < 0.25 as the threshold. The parameters that passed the first round of screening process included pre-treatment parameters including albumin (*P* = 0.09), albumin/globulin ratio (*P* = 0.22), neutrophil (*P* = 0.13), lymphocyte (*P* = 0.03), NLR (*P* = 0.01), dNLR (*p* = 0.13), and RBC (*p* = 0.23); early-on-/pre-treatment fold change parameters including LDH (*P* = 0.04), lymphocyte (*P* = 0.22), eosinophil (*P* = 0.13), and WBC (*P* = 0.17). Then, the optimal cut off value for the dichotomization of each of these continuous variables were calculated using the Youden Index. Details are listed in Supplementary Table 8.

These newly dichotomized variables (high vs. low) were then subjected to the second round of screening using the univariate logistic regression model, together with other clinicodemographical binary variables. Details are shown in Supplementary Table 9. Those with a *P* < 0.05 were thus incorporated into the multivariate analysis, which included pre-treatment NLR (threshold 2.24) and early-on-/pre-treatment LDH fold change (threshold 0.911). These two parameters were included in the same multivariate logistic model and confirmed to be independently correlated with best response. Details are listed in Supplementary Tables 9, 10.

### Risk Model for Best Response

Same screening criteria were adopted for the building of the best response (responder vs. non-responder) risk model. In total, three parameters were included in the risk model, including pre-treatment NLR, early-on-/pre-treatment LDH fold change, and M stage (with distant metastasis versus without). Same as in the survival risk model, each status which was negatively correlated with response scored 1, including pre-treatment NLR (>2.24), early-on/pre-treatment fold change of LDH (>0.911), and distant metastasis; otherwise, 0. Patients who scored 0, 1, 2, and 3 were referred to low, median-low, median-high, and high risk categories, respectively. The higher the risk category the patient belonged to, the more likely he/she was a non-responder to the anti-PD-1 monotherapy (OR 3.586, 95% CI, 1.668–7.713, *P* = 0.001). The objective response rate (ORR) of the patients with a low (*n* = 8), median-low (*n* = 18), median-high (*n* = 39), and high risk (*n* = 19) was 50.0% (95% CI, 15.7–84.3%), 27.8% (95% CI, 9.7–53.5%), 10.3% (95% CI, 2.9–24.2%), and 0%, respectively.

## Discussion

Due to the fact that there is a high degree of heterogeneity of response to the anti-PD-1 monotherapy between individual patients, there has been an unmet need with regard to the prognostic biomarker panel to guide therapeutic decision making ever since the dawn of immunotherapy. Although substantial efforts have been put into developing prognostic biomarkers, most of them are tumor sample and next generation sequencing-based (23). Also, there is a gradually increasing appreciation of the great discrepancy of response to the anti-PD-1 monotherapy between different melanoma subtypes, specifically acral and mucosal melanomas, which are predominant in Asian populations and have poorer responses compared with their cutaneous counterpart (24, 25) due to their distinct genomic and immunological makeups (22). Prognostic biomarker panels for Asian melanoma patients are still lacking. To address this clinically relevant issue, we performed an *ad hoc* analysis of two clinical trials testing anti-PD-1 monotherapy in China, and successfully built up three risk models for PFS, OS and response, respectively, facilitating clinical decision making.

Emerging data has demonstrated that the easily obtainable and highly clinically feasible parameters originated from generally tested complete blood cell and general chemistry tests are correlated with the clinical outcomes of advanced melanoma patients under immune checkpoint inhibitors, exemplified by the associations between a high NLR/dNLR/LDH, low eosinophil count, and poorer survival (15–20). However, to the best of our knowledge, although correlations have been well-delineated, there is no prognosis/best response-oriented biomarker panel developed yet. In this study, we made full use of easily accessible clinicodemographical data together with routinely tested biomarkers from both the complete blood cell and general chemistry tests and developed the first biomarker panels for both survival outcomes and best response, specifically for advanced Asian melanoma patient treated with the anti-PD-1 monotherapy that can facilitate clinical decision making.

In concert with previous observations, we observed negative correlations between NLR/dNLR/LDH and poorer survival outcomes (15–20), providing evidence that some of the previous reported peripheral blood prognostic markers in Caucasian populations are valid in Asian populations too.

By applying three rounds of a rigid screening process, we successfully selected the parameters that were most significantly correlated with survival outcomes and developed one simple and easily applicable risk model for PFS, OS, and best response each, which categorizes patients into low, median-low, median-high, and high risk subgroups, with a significantly decreasing PFS, OS, and ORR. According to our study design, all included parameters can be obtained before the second dose of anti-PD-1, which makes clinical therapeutic decision making more well-informed.

The major limitation of our study resides in 2-folds. First, due to the lack of a control cohort receiving targeted therapy or chemotherapy, we are not able to tell whether the risk model is prognostic or predictive. It can be the case that the patients categorized as low risk are the ones with a good physical performance, lower tumor burden, and indolent disease evolving trajectories. In addition, the patients with a high risk respond to all kinds of therapeutic modalities poorly. But still, the prognostic values of these models *per se* are informative, as they provide important information to answer the frequently asked prognosis-pertinent questions by patients in the routine clinical practice. Second, we acknowledge that this is a retrospective *ad hoc* study based on a cohort of patients with a limited sample size, and our findings have to be further validated by future prospective studies with a larger sample size to be adopted into the daily practice. As different anti-PD-1 agents are being approved in China, we believe that it is highly feasible to do so in the future. But for now, the complete lack of a risk model for advanced melanoma in the Asian populations and a great need for such models urge us to provide the so far most mature form of the model based on currently available data at hand serving as a momentum to inspire new thoughts and foster multicenter collaboration to finally achieve our eventual goals of putting forward a biomarker-based patient stratification and personalized precision medicine.

Taken together, we have developed risk models based on the easily accessible clinicodemographical characteristics and routinely tested laboratory test results that have well-distinguished the Asian advanced melanoma patients with different survival and best response outcomes treated with the anti-PD-1 monotherapy, providing important survival information for patients within different risk subcategories. For a broader implication, if successfully validated by future prospective study with a greater sample size, these risk models may serve as the criteria for patient stratification and there is a possibility that a personalized precision medicine in the era of immunotherapy could be based upon them.

## Data Availability Statement

The datasets presented in this article are not readily available because in order to protect the privacy of our patients, only deidentified information will be provided upon reasonable request in concordance to local regulatory requirements. Requests to access the datasets should be directed to silu15_silu@126.com.

## Ethics Statement

The studies involving human participants were reviewed and approved by Beijing Cancer Hospital Institutional Review Board. The patients/participants provided their written informed consent to participate in this study.

## Author Contributions

XB, JD, JG, and LS: concept and design, data analysis, and interpretation. XB, JD, CL, CC, LM, XWe, XS, ZC, XY, BT, BL, XWa, LZ, SL, YK, ZQ, HX, RD, JG, and LS: provision of study materials or patients. XB, JD, CL, JG, and LS: data collection and assembly. All authors: manuscript writing, editing, and final approval of manuscript.

## Conflict of Interest

XB has received a merit award supported by BMS. JG serves as consultant or is on advisory boards for MSD, Roche, Pfizer, Bayer, Novartis, Simcere Pharmaceutical Group, Shanghai Junshi Biosciences, and Oriengene. The remaining authors declare that the research was conducted in the absence of any commercial or financial relationships that could be construed as a potential conflict of interest.
