# Supplementary material for: Risk Models for Advanced Melanoma Patients Under Anti-PD-1 Monotherapy—Ad hoc Analyses of Pooled Data From Two Clinical Trials
Source: Front Oncol. 2021 May 20;11:639085. doi: 10.3389/fonc.2021.639085 (PMC8174451; doi:10.3389/fonc.2021.639085)
Supplement: Supplementary file 1 [file Data_Sheet_1.docx]

**Supplementary**

| **Characteristics** | **Number (%)** |
| --- | --- |
| Median age (range, year) | 53 (27-78) |
| Sex |  |
| Female | 45 (50.6) |
| Male | 44 (49.4) |
| Subtype |  |
| Cutaneous | 19 (21.3) |
| Acral | 37 (41.6) |
| Mucosal | 18 (20.2) |
| Ocular | 1 (1.1) |
| Unknown primary | 19 (21.3) |
| M stage |  |
| M1a | 33 (37.1) |
| M1b | 23 (25.8) |
| M1c | 27 (30.3) |
| M1d | 5 (5.6) |
| M0 | 1 (1.1) |
| BRAF |  |
| Mutant | 11 (12.4) |
| Wild type | 66 (74.2) |
| NA | 12 (13.5) |
| Prior treatment |  |
| Yes | 81 (91.0) |
| No | 8 (9.0) |
| Anti-PD-1 antibody |  |
| Pembrolizumab | 52 (58.2) |
| Camrelizumab | 37 (41.6) |

**Table S1. Clinicodemographical characteristics (n=89)**

**Table S2. First round screening for prognostic factors of PFS**

**(univariate Cox proportional hazard regression model)**

|  | **PFS** | | |
| --- | --- | --- | --- |
| **Parameters** | **HR (95% CI)** | **P value** | **Cutoff value** |
| **Clinicodemographical characteristics** |  |  |  |
| Age | 0.985 (0.967-1.003) | .09 | 55 |
| **Pre-treatment lab results** |  |  |  |
| LDH | 1.000 (0.999-1.001) | .80 |  |
| Total protein | 1.011 (0.989-1.058) | .63 |  |
| Albumin | 0.933 (0.874-0.996) | .04 | 42.5 |
| Globulin | 1.038 (0.995-1.084) | .09 | 24.2 |
| Albumin/globulin ratio | 0.569 (0.291-1.114) | .10 | 1.53 |
| Glucose | 1.125 (0.918-1.380) | .26 |  |
| Neutrophil | 1.153 (1.022-1.302) | .02 | 4.11 |
| Lymphocyte | 0.917 (0.624-1.348) | .66 |  |
| NLR | 1.102 (0.984-1.234) | .09 | 1.99 |
| dNLR | 90.02 (0.855-9477) | .06 | 0.9 |
| Monocyte | 2.014 (0.533-7.614) | .30 |  |
| Eosinophil | 1.365 (0.262-7.124) | .71 |  |
| Basophil | 2.820 (0.021-377) | .68 |  |
| WBC | 1.105 (0.991-1.233) | .07 | 4.34 |
| RBC | 1.027 (0.763-1.384) | .86 |  |
| Hb | 0.989 (0.977-1.001) | .06 | 153 |
| PLT | 1.004 (1.001-1.007) | .007 | 226 |
| **Fold change (early-on-/pre-treatment)** |  |  |  |
| LDH | 1.514 (0.762-3.007) | .24 | 1.259 |
| Total protein | 4.800 (0.082-281.5) | .45 |  |
| Albumin | 5.437 (0.112-263.6) | .39 |  |
| Globulin | 0.871 (0.179-4.247) | .86 |  |
| Albumin/globulin ratio | 1.271 (0.233-6.940) | .78 |  |
| Glucose | 0.464 (0.095-2.259) | .34 |  |
| Neutrophil | 0.918 (0.461-1.827) | .81 |  |
| Lymphocyte | 0.705 (0.296-1.682) | .43 |  |
| NLR | 1.263 (0.690-2.311) | .45 |  |
| dNLR | 0.185 (0.010-3.439) | .26 |  |
| Monocyte | 0.964 (0.520-1.789) | .91 |  |
| Eosinophil | 0.790 (0.622-1.003) | .05 | 1.176 |
| Basophil | 0.973 (0.926-1.023) | .29 |  |
| WBC | 0.924 (0.31-2.578) | .88 |  |
| RBC | 0.300 (0.044-2.027) | .22 | 0.940 |
| Hb | 0.420 (0.012-14.160) | .63 |  |
| PLT | 1.271 (0.395-4.090) | .69 |  |

**Table S3. Univariate Cox Regression model for dichotomized and binary variables for PFS**

|  | **PFS** | |
| --- | --- | --- |
| **Parameters** | **HR (95% CI)** | **P value** |
| **Clinicodemographical** |  |  |
| Sex | 0.808 (0.521-1.252) | .34 |
| Melanoma subtype (non-cutaneous) | 1.005 (0.587-1.721) | .99 |
| Prior treatment (with vs. without) | 1.120 (0.515-2.437) | .77 |
| BRAF | 1.537 (0.801-2.949) | .20 |
| Age (>55) | 0.600 (0.381-0.943) | .03 |
| **Pre-treatment** |  |  |
| Albumin (>42.5g/L) | 0.453 (0.274-0.750) | .002 |
| Globulin (>24.2 g/L) | 1.606 (0.953-2.708) | .08 |
| Albumin/globulin ratio (>1.53) | 0.658 (0.422-1.026) | .06 |
| Neutrophil (>4.11*10^9^/L) | 2.082 (1.291-3.357) | .003 |
| NLR (>1.99) | 1.823 (1.126-2.951) | .01 |
| dNLR (>0.9) | 1.760 (1.078-2.873) | .02 |
| WBC (>4.34*10^9^/L) | 1.765 (0.997-3.124) | .05 |
| Hb (>153g/L) | 0.410 (0.188-0.896) | .03 |
| PLT (>226*10^9^/L) | 1.951 (1.241-3.069) | .004 |
| **Fold change (early-on/pre-treatment)** |  |  |
| LDH (>1.259) | 1.558 (0.920-2.638) | .10 |
| Eosinophil (>1.176) | 0.562 (0.353-0.895) | .02 |
| RBC (>0.940) | 0.533 (0.317-0.896) | .02 |

**Table S4. Multivariate Cox proportional hazard regression model for PFS**

| **Parameter** | **HR (95% CI)** | **P value** |
| --- | --- | --- |
| **Clinicodemographical** |  |  |
| Age (>55 y.o.) | 0.692 (0.417-1.150) | .16 |
| **Pre-treatment** |  |  |
| Albumin (>42.5g/L) | 0.653 (0.357-1.220) | .17 |
| Neutrophil (>4.11*10^9^/L) | 1.603 (0.948-2.708) | .08 |
| NLR (>1.99) | 1.217 (0.712-2.080) | .47 |
| dNLR (>0.9) | 1.832 (0.997-3.365) | .05 |
| Hb (>153g/L) | 0.356 (0.151-0.843) | .02 |
| PLT (>226*10^9^/L) | 1.406 (0.840-2.353) | .19 |
| **Fold change (early-on-/pre-treatment)** |  |  |
| Eosinophil (>1.176) | 0.500 (0.306-0.816) | .006 |
| RBC (>0.940) | 0.676 (0.378-1.209) | .19 |

**Table S5. First round screening for prognostic factors of OS**

**(univariate Cox proportional hazard regression model)**

|  | **OS** | | |
| --- | --- | --- | --- |
| **Parameters** | **HR (95% CI)** | **P value** | **Cutoff value** |
| **Clinicodemographical characteristics** |  |  |  |
| Age | 0.997 (0.974-1.021) | .80 |  |
| **Pre-treatment lab results** |  |  |  |
| LDH | 1.001 (1.000-1.002) | .20 | 305 |
| Total protein | 1.005 (0.957-1.055) | .86 |  |
| Albumin | 0.982 (0.906-1.065) | .66 |  |
| Globulin | 1.001 (0.960-1.043) | .97 |  |
| Albumin/globulin ratio | 0.804 (0.371-1.741) | .58 |  |
| Glucose | 1.028 (0.765-1.382) | .86 |  |
| Neutrophil | 1.287 (1.087-1.524) | .003 | 5.16 |
| Lymphocyte | 1.019 (0.630-1.650) | .94 |  |
| NLR | 1.191 (1.037-1.367) | .01 | 4.33 |
| dNLR | 3.287 (0.012-894.4) | .68 |  |
| Monocyte | 3.615 (0.643-20.340) | .15 | 0.47 |
| Eosinophil | 5.517 (0.687-44.310) | .11 | 0.09 |
| Basophil | 1.949 (0.033-114.800) | .75 |  |
| WBC | 1.211 (1.044-1.405) | .01 | 4.29 |
| RBC | 1.167 (0.747-1.823) | .50 |  |
| Hb | 0.991 (0.976-1.007) | .28 |  |
| PLT | 1.003 (1.000-1.006) | .04 | 226 |
| **Fold change (early-on-/pre-treatment)** |  |  |  |
| LDH | 2.581 (1.022-6.522) | .04 | 0.911 |
| Total protein | 1.440 (0.006-350.6) | .89 |  |
| Albumin | 0.313 (0.002-45.230) | .65 |  |
| Globulin | 3.116 (0.390-24.890) | .28 |  |
| Albumin/globulin ratio | 0.288 (0.026-3.202) | .31 |  |
| Glucose | 1.031 (0.130-8.195) | .98 |  |
| Neutrophil | 2.122 (0.778-5.788) | .14 | 1.539 |
| Lymphocyte | 0.974 (0.351-2.698) | .96 |  |
| NLR | 1.486 (0.694-3.182) | .31 |  |
| dNLR | 0.023 (0-4.760) | .17 | 0.935 |
| Monocyte | 1.753 (0.772-3.983) | .18 | 1.529 |
| Eosinophil | 1.009 (0.747-1.363) | .96 |  |
| Basophil | 0.955 (0.855-1.066) | .41 |  |
| WBC | 2.516 (0.610-10.380) | .20 | 1.116 |
| RBC | 0.536 (0.082-3.501) | .52 |  |
| Hb | 1.241 (0.006-263.8) | .94 |  |
| PLT | 1.491 (0.344-6.451) | .59 |  |

**Table S6. Univariate Cox Regression model for dichotomous/categorical variables for OS**

| **Parameters** | **HR (95% CI)** | **P value** |
| --- | --- | --- |
| **Clinicodemographical** |  |  |
| Sex | 1.108 (0.628-1.954) | .72 |
| Melanoma subtype (non-cutaneous) | 0.708 (0.365-1.374) | .31 |
| Prior treatment (with vs. without) | 1.345 (0.417-4.341) | .62 |
| BRAF | 0.724 (0.284-1.844) | .50 |
| **Pre-treatment** |  |  |
| LDH (>305U/L) | 1.769 (0.960-3.261) | .07 |
| Neutrophil (>5.16*10^9^/L) | 3.921 (1.900-8.092) | <.001 |
| NLR (>4.33) | 2.866 (1.374-5.977) | .005 |
| Monocyte (>0.47*10^9^/L) | 2.174 (1.159-4.080) | .02 |
| Eosinophil (>0.09*10^9^/L) | 2.108 (1.163-3.823) | .01 |
| WBC (>4.29*10^9^/L) | 3.050 (1.094-8.503) | .03 |
| PLT (>226*10^12^/L) | 2.529 (1.413-4.528) | .002 |
| **Fold change (early-on-/pre-treatment)** |  |  |
| LDH (>0.911) | 3.053 (1.349-6.910) | .007 |
| Neutrophil (>1.539) | 2.458 (1.135-5.323) | .02 |
| DNLR (>0.935) | 0.528 (0.235-1.189) | .12 |
| Monocyte (>1.529) | 1.726 (0.801-3.718) | .16 |
| WBC (>1.116) | 1.736 (0.949-3.173) | .07 |

**Table S7.** **Multivariate Cox proportional hazard regression model for OS**

| **Parameters** | **HR (95% CI)** | **P value** |
| --- | --- | --- |
| **Pre-treatment** |  |  |
| Neutrophil (>5.16*10^9^/L) | 3.186 (1.019-9.965) | .05 |
| NLR (>4.33) | 2.356 (0.741-7.488) | .15 |
| Monocyte (>0.47*10^9^/L) | 0.833 (0.741-7.488) | .64 |
| Eosinophil (>0.09*10^9^/L) | 2.123 (1.065-4.233) | .03 |
| WBC (>4.29*10^9^/L) | 2.095 (0.617-7.112) | .24 |
| PLT (>226*10^9^/L) | 1.971 (0.969-4.009) | .06 |
| **Fold change (early-on-/pre-treatment)** |  |  |
| LDH (>0.911) | 3.135 (1.232-7.977) | .02 |
| Neutrophil (>1.539) | 2.216 (0.951-5.164) | .07 |

**Table S8. First round screening for predictive factors of non-responder**

**(univariate logistic regression model)**

|  | **Non-responder** | | |
| --- | --- | --- | --- |
| **Parameters** | **OR (95% CI)** | **P value** | **Cutoff value** |
| **Clinicodemographical characteristics** |  |  |  |
| Age | 0.988 (0.943-1.035) | .62 |  |
| **Pre-treatment lab results** |  |  |  |
| LDH | 1.001 (0.997-1.005) | .54 |  |
| Total protein | 1.000 (0.900-1.111) | >.99 |  |
| Albumin | 0.834 (0.675-1.030) | .09 | 49.4 |
| Globulin | 1.053 (0.937-1.184) | .38 |  |
| Albumin/globulin ratio | 0.364 (0.073-1.815) | .22 | 2.49 |
| Glucose | 1.078 (0.599-1.939) | .80 |  |
| Neutrophil | 1.488 (0.885-2.502) | .13 | 4.27 |
| Lymphocyte | 0.298 (0.102-0.871) | .03 | 2.15 |
| NLR | 3.540 (1.332-9.405) | .01 | 2.24 |
| dNLR | 2560 (0-7.362*10^7^) | .13 | 0.90 |
| Monocyte | 1.058 (0.035-31.994) | .97 |  |
| Eosinophil | 27.892 (0.038-20360) | .32 |  |
| Basophil | 0.906 (0-53023) | .99 |  |
| WBC | 1.114 (0.795-1.560) | .53 |  |
| RBC | 1.698 (0.710-4.060) | .23 | 4.21 |
| Hb | 0.979 (0.945-1.016) | .26 |  |
| PLT | 1.004 (0.995-1.012) | .40 |  |
| **Fold change (early-on-/pre-treatment)** |  |  |  |
| LDH | 18.751 (1.157-304.005) | .04 | 0.911 |
| Total protein | 267.368 (0.004-1.712*10^7^) | .32 |  |
| Albumin | 9.100 (0-1.707*10^5^) | .66 |  |
| Globulin | 9.538 (0.042-2.161*10^3^) | .42 |  |
| Albumin/globulin ratio | 0.276 (0.002-38.648) | .61 |  |
| Glucose | 0.812 (0.013-50.437) | .92 |  |
| Neutrophil | 2.236 (0.357-13.998) | .39 |  |
| Lymphocyte | 5.410 (0.363-80.654) | .22 | 1.106 |
| NLR | 1.035 (0.258-4.150) | .96 |  |
| dNLR | 0.010 (0-55.672) | .29 |  |
| Monocyte | 1.436 (0.256-8.036) | .68 |  |
| Eosinophil | 0.663 (0.392-1.123) | .13 | 0.250 |
| Basophil | 0.891 (0.726-1.094) | .27 |  |
| WBC | 6.787 (0.429-107.442) | .17 | 1.137 |
| RBC | 0.132 (0.004-4.371) | .26 |  |
| Hb | 0.736 (0-6113) | .95 |  |
| PLT | 1.293 (0.078-21.536) | .86 |  |

**Table S9. Univariate Logistic Regression model for dichotomized and binary variables for best response (responder vs. non-responder)**

|  | **Non-responder** | |
| --- | --- | --- |
| **Parameters** | **OR (95% CI)** | **P value** |
| **Clinicodemographical** |  |  |
| Sex | 2.314 (0.652-8.210) | .19 |
| Melanoma subtype (non-cutaneous) | 0.577 (0.116-2.867) | .50 |
| Prior treatment (with vs. without) | NA | .99 |
| Mutation (BRAF) | 0.882 (0.165-4.713) | .88 |
| **Pre-treatment** |  |  |
| Albumin (>49.4g/L) | NA | .99 |
| Albumin/globulin ratio (>2.49) | NA | .99 |
| Neutrophil (>4.27*10^9^/L) | 5.288 (0.659-44.033) | .12 |
| Lymphocyte (>2.15*10^9^/L) | 0.902 (0.173-4.687) | .90 |
| NLR (>2.24) | 15.484 (1.909-125.585) | .01 |
| dNLR (>0.9) | NA | .99 |
| **Fold change (early-on-/pre-treatment)** |  |  |
| LDH (>0.911) | 6.514 (1.846-22.990) | .004 |
| Lymphocyte (>1.106) | 3.581 (0.738-17.388) | .11 |
| Eosinophil (>0.250) | NA | .99 |
| WBC (>1.137) | 6.522 (0.801-53.081) | .08 |

*NA, not available.

**Table S10. Multivariate logistic regression model for best response**

|  | **Non-responder** |  |
| --- | --- | --- |
| **Parameter** | **OR (95% CI)** | **P value** |
| **Pre-treatment** |  |  |
| NLR (>2.24) | 16.664 (1.926-144.146) | .01 |
| **Fold change (early-on-/pre-treatment** |  |  |
| LDH (>0.911) | 7.086 (1.761-28.514) | .006 |
